# Strategies to Improve the Integration of Community Health Workers Into Health Care Teams: “A Little Fish in a Big Pond”

**DOI:** 10.5888/pcd12.150199

**Published:** 2015-09-17

**Authors:** Caitlin G. Allen, Cam Escoffery, Anamika Satsangi, J. Nell Brownstein

**Affiliations:** Author Affiliations: Cam Escoffery, Anamika Satsangi, J. Nell Brownstein, Emory University, Rollins School of Public Health, Atlanta, Georgia.

## Abstract

**Introduction:**

The Patient Protection and Affordable Care Act acknowledges the value of community health workers (CHWs) as frontline public health workers. Consequently, growing attention has been placed on promoting CHWs as legitimate partners to provide support to health care teams and patients in the prevention, management, and control of chronic disease, particularly among diverse populations and high-need individuals.

**Methods:**

Using a mixed-methods research approach, we investigated the integration of CHWs into health care teams from the CHW perspective. We conducted a survey of 265 CHWs and interviews with 23 CHWs to better understand and describe their experience and their perceived opportunities and challenges regarding their integration within the context of health care reform.

**Results:**

Feelings of organizational support were positively correlated with the number of CHWs in the organization. CHWs reported the following facilitators to integration: having team meetings (73.7%), training inside (70.4%) and outside of the organization (81.6%), access to electronic health records, and ability for CHWs to stay connected to the community.

**Conclusion:**

The perspectives of CHWs on their positive and negative experiences offer useful and innovative insight into ways of maximizing their impact on the health care team, patients, and their role as key emissaries between clinical services and community resources.

## Introduction

Although community health workers (CHWs) have been active in the United States for more than 6 decades (1), it is not until recently that they have been considered as legitimate partners to provide support to health care teams in the prevention, management, and control of chronic disease, particularly for diverse populations and for high-need individuals. Since the enactment of the Patient Protection and Affordable Care Act (PPACA) in 2010, much attention has been placed on this unique workforce. As members of the community they serve, CHWs are effective at bridging the gap between health care and the community by providing culturally and linguistically appropriate health education, offering follow-up care, and conducting case management and care coordination for people with complex needs (2–5). CHWs are poised to enter the mainstream of health care, and the PPACA offers an exceptional platform from which to integrate CHWs into primary care and prevention efforts (6–8).

Despite meaningful efforts, CHWs have largely been excluded from the health care system because of funding and reimbursement issues (6). The PPACA allows the incorporation of CHWs into health care teams. CHWs have also been cited as natural candidates for ambulatory care practices working toward patient-centered medical homes (PCMHs) (9). Although many stakeholders are interested in and pursuing models that incorporate CHWs as care team members, research on CHW integration is limited (3,10), and no widely accepted evidence-based techniques exist for integrating CHWs into health care settings. Furthermore, current research is limited on CHWs’ own perspectives on their roles and on their integration into health care teams. The objective of this study was to provide insight on the challenges and the support required for optimal CHW integration and answer the question, how are CHWs integrated into their health care organization?

## Methods

All aspects of this study were approved by the Emory University Institutional Review Board. A total of 265 CHWs participated in the cross-sectional survey. CHWs were recruited through an email listserv sponsored by the American Public Health Association (APHA) of 30 CHW networks and associations in 19 states. The first phase of the study included an online survey using Survey Monkey consisting of 56 questions. In this study, we conducted a subanalysis of the data on CHWs’ roles and integration of CHWs into health care teams. Survey responses included multiple-choice, open-ended, and Likert-scale options. Word choice and sentence construction of survey questions were at an eighth-grade reading level (according to the Flesh–Kincaid readability test), and questions were pilot tested with 4 CHWs to ensure that all participants had a clear understanding. The survey was open from August through October 2014. The beginning of the survey included the APHA definition of CHWs, and participants were asked to self-identify as a CHW before beginning. Data were imported from Survey Monkey into SPSS Statistics version 22 (11) and included descriptive and bivariate statistics. In addition to other questions, CHWs were asked to rate their level of agreement on a 5-point Likert scale with 6 statements about support they receive for their work (1, strongly disagree; 2, disagree; 3, neutral; 4, agree; 5, strongly agree). Responses to these questions were summed to create a composite score for each respondent and used to analyze correlations between satisfaction with integration with the health care team and various aspects of the organization (eg, number of CHWs working in the organization) and CHW characteristics (eg, amount of time working as a CHW). We used Spearman rank correlation (ρ) to determine correlations.

We also conducted 23 semistructured qualitative interviews with CHWs from 17 states and the District of Columbia. At the end of the survey, respondents were asked if they would be willing to participate in an interview; willing respondents were contacted if they answered more than half of the survey questions. The interview guide consisted of 21 open-ended questions with probes that aligned with the survey content (Box). Interviews lasted an average of 53.5 minutes. The principal investigator received informed verbal consent before the interview and also received permission to record the interview. The interview was conducted over the telephone, recorded, and transcribed verbatim. Interviews were quality controlled; a codebook was developed and adapted on completion of a consensus review between the 2 primary coders (C.G.A. and A.S.). The codes were compiled in MAXQDA version 11 (12) and retrieved individually. Researchers used inductive thematic analysis for qualitative analysis, a common form of data analysis that involves reading transcribed interviews, identifying themes, coding, and interpreting the content of these codes and themes (13). This technique included careful reading for themes and further subcoding or classification. Qualitative and quantitative data were combined through triangulation techniques (14).

**Box. Sample Interview Questions^a^ on Organizational Implementation, Study on Improving the Integration of Community Health Workers (CHWs) Into Health Care Teams, 2014**

**Table:** 

Quantitative Survey	Qualitative Interview
1. Please indicate how much you agree with the following statement: I feel well supported by my organization (eg, supervisor, providers, other team members) in carrying out my duties as a CHW. [Strongly agree, Agree, Neutral, Disagree, Strongly Disagree]	1. How are CHWs treated/viewed in your organization?
2. How hard or easy has it been for you to become part of your organization’s care team? [Very easy, Easy, Neither/either easy or hard, Hard, Very hard]	2. What within your organization helps you do your job as a CHW?
3. Please indicate how much you agree with the following statement: My organization will continue to support my work and the work of others as a CHW in the future. [Strongly agree, Agree, Neutral, Disagree, Strongly disagree]	3. What about your organization gets in the way of your doing your job?
	4. How do other team members (clerical staff, doctors, nurses, social workers, medical assistants, etc.) interact with you or other CHWs?

^a ^Questions were generated from literature review of previous CHW surveys, the Consolidated Framework for Implementation Research integration theory (15), and expert consultation.

## Results

Participants came from diverse backgrounds across the United States (Table 1). CHWs worked with people through individual telephone or email sessions (64.2%), home visits (68.6%), outreach in the organization setting (71.1%), outreach in the community setting (73.0%), and sessions within the organization (74.5%). The most frequently reported roles were helping people gain access to medical services (86.9%), advocating for individual needs (86.9%), teaching people how to use health care and social services (78.2%), helping people gain access to nonmedical services (78.2%), and helping people manage chronic conditions (77.2%). Besides working with patients, nearly half of CHWs reported working with nurses (49.7%), physicians (46.2%), or other CHWs (43.4%).

**Table 1:** Selected Demographic Information of Survey Participants (n = 265), Study on Improving the Integration of Community Health Workers (CHWs) Into Health Care Teams, 2014^a^^,^ b

Characteristic	Value^a^
**Age, mean (SD), y**	43.1 (12.8)
**Race**	
No. of respondents	160
American Indian/Alaskan Native	8 (5.0)
Asian/Pacific Islander	2 (1.2)
Black/African American	39 (24.4)
Hispanic/Latino(a)	69 (43.1)
Non-Hispanic white	38 (23.8)
Other race/ethnicity	4 (2.5)
**Sex**	
No. of respondents	154
Female	136 (88.3)
**Highest grade of school**	
No. of respondents	153
8th grade or less	0
Some high school	0
High school or GED	22 (14.4)
Some college or technical	48 (31.4)
College graduate	59 (38.6)
Post-graduate or professional	24 (15.7)
**Census region**	
No. of respondents	198
Region 1 (Northeast)	22 (11.1)
Region 2 (Midwest)	79 (39.9)
Region 3 (South)	50 (25.3)
Region 4 (West)	47 (23.7)
**Organization type**	
No. of respondents	204
Clinic (not a FQHC)	24 (11.8)
Community-based organization	53 (26.0)
FQHC	37 (18.1)
Health insurance company	3 (1.5)
Hospital	32 (15.7)
Local health department	22 (10.8)
Indian health service	1 (0.5)
Tribal health department	5 (2.5)
Urban health center	1 (0.5)
University	8 (3.9)
Nonprofit	4 (2.0)
Non-university school system	6 (2.9)
Other	8 (3.9)
**Years as a CHW and at organization**	
No. of respondents	205
Years as CHW, median (interquartile range)	4.0 (1.4–12.0)
Years at organization, median (interquartile range)	4.0 (1.0–10.0)

Abbreviations: FQHC, federally qualified health center; GED, general education development; SD, standard deviation.

a Values are number of respondents (percentage) unless otherwise indicated.

b Percentages are based on the number of respondents to question.

Most survey respondents agreed or strongly agreed with statements on organizational support (Table 2), and most (84.4%) felt that their organization would continue to support their work.

**Table 2:** Responses to Survey Questions^a^ on Organizational Support for CHWs, Study on Improving the Integration of Community Health Workers (CHWs) Into Health Care Teams, 2014^b^

Statement	Strongly Disagree	Disagree	Neutral	Agree	Strongly Agree	Mean Score (SD)
I feel well supported by my organization in carrying out my duties as a CHW	15 (9.6)	8 (5.1)	12 (7.7)	57 (35.5)	64 (41.0)	3.9 (1.3)
People put a lot of effort into making CHWs a success at my organization	16 (10.4)	9 (5.8)	24 (15.5)	51 (32.9)	55 (35.5)	3.8 (1.3)
People at my organization believe CHWs are important	14 (9.1)	9 (5.8)	20 (13.0)	50 (32.5)	61 (39.6)	3.9 (1.3)
Managers and supervisors at my organization are strongly committed to working with CHWs	15 (9.7)	5 (3.2)	22 (14.2)	57 (36.8)	56 (36.1)	3.9 (1.2)
I am part of my organization’s care team for patients or clients	6 (4.0)	4 (2.6)	22 (14.6)	58 (38.4)	61 (40.4)	4.1 (1.0)
My organization will continue to support my work and the work of other CHWs in the future	9 (5.8)	1 (.6)	14 (9.1)	57 (37.0)	73 (47.4)	4.2 (1.0)

Abbreviations: SD, standard deviation

a CHWs were asked to rate their level of agreement with statements about support they receive for their work on a 5-point Likert scale, with strongly agree = 5, agree = 4, neutral = 3, disagree = 2, and strongly disagree = 1.

b Values are number of respondents (percentage) unless otherwise indicated.

Thirty-seven percent of CHWs selected neutral when asked, “How hard or easy has it been for you to become part of your organization’s care team?” The mean score for this question was 2.6 (standard deviation [SD], 1.0). Qualitatively, several CHWs indicated difficulty with becoming a part of the care team. Integration took time and required building trust between the CHW and other members of the team. Lack of knowledge about how to work with CHWs was a barrier to the CHWs becoming integrated. For example:

I’m put here in the office and I’m given a list, but we don’t thrive that way because . . . we’re from the community. We’re like the last man on the totem pole, so to speak, so it takes a lot of support. It takes a lot of backing up, informing the immediate staff that I work with, so they know how to utilize me, making sure that they’re on board and being more supportive and including me in the health care plan instead of leaving me just as an option in an office.

### Characteristics of successful CHW organizations

The 6-item organizational satisfaction composite score ranged from 6 (strongly disagree) to 30 (strongly agree). Respondents had a mean score of 23.8 (SD, 5.5). The Cronbach α for the 6-item scale was 0.87.

#### Number of CHWs

The number of CHWs in an organization was positively correlated with the organizational satisfaction composite score (ρ = 0.24, *P* = .02, n = 105) (Figure). Further breakdown of the 6-item scale showed that feelings of “People put a lot of effort into making CHWs a success at my organization” was positively correlated with the number of CHWs in the organization (ρ = 0.22, *P* = .02, n = 114). Forty-three percent of CHWs reported working with other CHWs; we found an average of 9.6 CHWs per organization (SD, 10.6).

**Figure:**
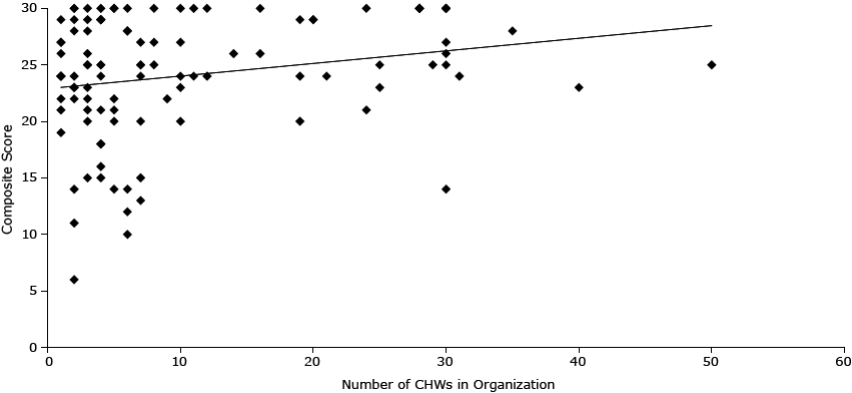
Correlation of number of CHWs in an organization and satisfaction with the way CHWs are integrated into the health care team of an organization. **Table:** 

Number Community Health Workers	Composite Score
19	20
14	26
5	30
3	23
6	14
16	26
4	18
5	30
24	30
19	29
1	22
1	27
2	24
8	25
7	13
2	6
7	20
8	30
7	15
7	27
7	25
6	10
7	24
1	24
4	25
7	25
3	26
3	29
2	14
5	21
4	18
3	15
2	30
10	30
4	29
1	26
12	24
2	22
2	30
4	16
9	22
4	29
28	30
4	30
12	30
1	21
10	23
1	19
2	23
3	22
25	25
1	24
10	20
4	15
30	27
2	23
21	24
35	28
28	30
20	29
4	24
10	27
2	29
8	27
3	21
40	23
6	28
30	25
3	25
2	28
3	20
3	28
6	12
4	29
1	29
10	24
3	30
1	24
11	30
11	24
4	21
5	20
6	28
5	22
4	25
50	25
25	23
1	27
30	30
3	25
30	14
16	30
20	29
2	11
6	30
29	25
30	30
30	26
5	14
24	21
30	30
31	24
28	30
19	24

Also positively correlated with the number of CHWs in the organization were perceptions of being a part of the care team (ρ = 0.33, *P* = .001, n = 111) and feelings that the organization will continue to support CHWs (ρ = 0.20, *P* = .04, n = 114).

#### CHW networking

As a factor that supports their work, 77.9% of CHWs cited networking with other CHWs, 50.3% cited networking with non-CHW organizations, and 49.7% cited being a member of a CHW alliance or association. Connecting with other CHWs allowed CHWs to do their job, offer technical assistance to each other, and gain access to the patients with the greatest need. For example:

There are a lot of amazing CHWs here . . . when I find a particular need that I don’t know what to do, I know who to go to so that person can come in and help me with that patient to get them where they need to be . . . even though it’s not a recognized networking system yet, there is a huge community bonding here.

#### Team meetings

Staff meetings were an important component of organizations that allowed CHWs to feel supported in their role. Seven in ten (73.7%) CHWs cited attendance at staff meetings as a facilitator for their work. Meetings included large organizational meetings (eg, executive-level meetings), nontraditional meetings designed to help team members understand the day-to-day functions of CHWs (eg, taking supervisors to site visits, health fairs), and one-on-one case management and care coordination meetings. Regardless of the type of meeting, CHWs appreciated having consistent meetings that were run with respect and prioritized CHW’s knowledge of the community. These meetings assisted with demonstrating the value added by CHWs and provided an opportunity for CHW recognition and sharing. For example:

We have staff meetings every week and the whole team is there at the primary care practice. . . . I think that’s great because it gets everybody to sit down, and at the beginning of the staff meeting we talk, do shout-outs and stories, and people can go around, and they share great experiences they’ve had, whether big or small.

#### Electronic health records

Although we did not ask CHWs about electronic health records (EHRs) in the survey, most interviewees discussed EHRs or other tracking methods as an important component of their work. According to interviewees, EHRs helped to integrate CHWs into the care team and were used to make appointments and communicate directly and indirectly with providers. EHRs helped providers track patients while also acknowledging the value added by CHWs.

#### Training

In the survey, CHWs reported attending both training held outside of the organization (81.6%) and training held in the organization (70.4%) as important in helping them do their work. Training style, length, and need varied widely by organizational setting, but in general CHWs sought well-organized, comprehensive, clear training and training standards. Interview participants actively sought further training and continuing education. Some CHWs had participated in disease-specific training, and those who did not expressed a desire for it. Qualitative interviews showed that CHWs considered life experience and being part of their community as important features of their training and being appropriately prepared for their role. The following quote from a clinic-based CHW demonstrates the importance of life experience: “I’ve done a lot of different trainings. I think it’s honestly life experience [that] really prepared me for this role more than anything, and then once I got to the job, I did so much research and digging into what is a CHW, to make sure I’d be the best one.”

Overall, CHWs recognized the importance of high-quality, continuous training and supervision by clinical and nonclinical staff, which helps ensure the fidelity of information delivered to patients and evidence-based practice. For example, one interviewee said, “If you provide adequate training for folks, then you facilitate both what they’re able to do and how useful it is for the community, and it takes a lot of the problems out of the things. It puts parameters on it.”

One CHW summarized her experience in the organization. Even with support from staff members, she described herself as just one part of the overall organization.

I feel like my voice is heard but . . . it’s like I’m a little fish in a big pond because there are so many other things that they’re focusing on right now, that sometimes my role and position gets put on the back burner. I think that’s the biggest issue that I’m having here, now, is that they adopted the concept here — it’s a great concept, but my role won’t thrive unless I have the support that I need.

## Discussion

Multidisciplinary care teams and interprofessional collaboration are critical to the success of health care reform. CHWs are well suited to join care teams to address health disparities and social determinants of health. Previous studies about CHW integration emphasize similar themes found in this study, including workflow, communication, and EHR use (16). This research is unique because it presents CHWs’ perspective about their roles and their integration into health care teams as they relate to the opportunities provided by the PPACA.

Throughout this study, CHWs described important facilitators to care-team integration, including maintaining connection and support from other CHWs. Because CHWs are particularly social, creative, and well-connected individuals within social networks, supporting this important facilitator will enhance CHW contributions to patients and members of the care team as well as the community. Thus, we recommend hiring CHWs who are members of or have a close understanding of the community, shared life experience, and desire to help the community (2,5,17,18). We also recommend ongoing job support or joining professional associations for CHWs to connect with each other.

Another facilitator to CHW integration is including CHWs in well-run, consistent, organized meetings. Case management meetings involving CHWs allow the care team to understand critical pathways and issues patients face outside of the health care setting and facilitate the exchange of information to help build cases or understanding of patients (19). Learning these pathways also allows the health care team to tailor their educational information for high-risk and high-need patients. Case management and meetings with CHWs help to improve provider engagement with patients by encouraging them to take a more active role and assume responsibility in chronic disease management, encouraging collaboration, and helping to increase the CHW’s sense of autonomy. Communication is beneficial to the well-being of the CHW and to the care team and patient.

Communication can be facilitated by appropriate workflows, training, and EHRs (3,20–22). EHRs and other databases, when used correctly, are cited in this study and in previous studies as excellent tools for tracking CHW–patient interactions and communicating within teams to provide continuous care (21,23). EHRs allow organizations to build systems that integrate social data with clinical data, creating a powerful new tool to move the “comprehensive population health agenda forward” (22). The analytic capacity of an organization dictates its functional and organizational capacity, which can lead to potential funding mechanisms. Proficient use of EHRs can not only track and improve patient outcomes but also make the business case for the value of CHWs as members of care teams and is critical to understanding the role and integration of CHWs in health systems. Our study calls for investments by health care organizations in training (requested and needed by CHWs), team building, and regular two-way communication between CHWs and health care providers (24).

A limitation of this study is its focus solely on gaining CHWs’ perspectives on their integration into health care teams. Other studies included the perspectives of non-CHW members (25). One study surveyed providers, who unanimously agreed that the CHWs’ work with patients on health education and patient follow-up allowed them to work more efficiently, enhanced the care for patients, and freed up provider time (8). Previous research about CHWs revealed a paradox between the ways CHWs perceived their work and the ways that the employing organization perceived CHWs’ work. For example, CHWs perceived their work to be locally focused, whereas the employers were expanding the geographic scope of work outside of the community served because of demand (26). Such a paradox undermines the working environment of CHWs and calls for a balance that will help sustain the relationship between the organization and CHWs. Future studies could explore all team members’ perspectives of CHW integration in an effort to better understand feelings of support, patient care coordination, and integration. Another limitation is that CHWs who self-selected to participate in the survey and interview may not be representative of all CHWs who serve in communities. Our sample reported being highly connected to other CHWs and having computer access.

Because of the opportunities and promising models emerging for CHWs in the context of the PPACA, research should continue to focus on best methods to measure CHW integration; develop evidence-based methods for CHW integration; explore key roles CHW may play in PPACA from multiple perspectives (eg, patients and care team); make core training recommendations that could lead to further recognition of CHWs and cost reimbursement for their services; and develop rigorous cost effectiveness protocols to measure CHW efforts in disease prevention and management.

Ongoing support of the PPACA and provisions such as the PCMH will continue to improve health outcomes through restructuring and redefining the health care system to focus on holistic, preventive, patient-centered efforts. Support (ie, financing and reimbursement) and incentives for the PCMH model and CHWs should be included to advance the mission of the PPACA and to reduce health disparities and increase health equity. CHWs are uniquely situated between public health and health care and have a particularly important role in representing the communities served. With the PPACA’s focus on public health, CHWs are best suited to bridge the gap and strengthen relationships between primary care providers and community members; however, without careful thought and consideration, CHWs could get lost in the health care system, which could have an adverse effect on patient-centered care (27,28). Communicating closely, advocating for inclusion of the CHW perspective, promoting the integration of CHWs in the full range of health care delivery and population health programs (27), building staff support (29), recognizing CHW contributions (3), and working respectfully (30) with CHWs throughout the implementation of the PPACA and patient-centered care will allow organizations to maximize their impact and create sustainable new models of care.

## References

[1] US Department of Health and Human Services. Community health worker national workforce study. http://bhpr.hrsa.gov/healthworkforce/reports/chwstudy2007.pdf. Accessed May 20, 2015.

[2] Rosenthal EL , Wiggins N , Brownstein JN , Johnson S , Borbon IA , Rael R . The final report of the National Community Health Advisor Study: weaving the future. Tuscon (AZ): University of Arizona; 1998.

[3] Findley S , Matos S , Hicks A , Chang J , Reich D . Community health worker integration into the health care team accomplishes the triple aim in a patient-centered medical home: a Bronx tale. J Ambul Care Manage 2014;37(1):82–91. 24309397 10.1097/JAC.0000000000000011

[4] Peretz PJ , Matiz LA , Findley S , Lizardo M , Evans D , McCord M . Community health workers as drivers of a successful community-based disease management initiative. Am J Public Health 2012;102(8):1443–6. 10.2105/AJPH.2011.300585 22515859 PMC3464827

[5] Gilkey M , Garcia CC , Rush C . Professionalization and the experience-based expert: strengthening partnerships between health educators and community health workers. Health Promot Pract 2011;12(2):178–82. 10.1177/1524839910394175 21427271

[6] Martinez J , Ro M , Villa NW , Powell W , Knickman JR . Transforming the delivery of care in the post-health reform era: what role will community health workers play? Am J Public Health 2011;101(12):e1–5. 10.2105/AJPH.2011.300335 22021289 PMC3222444

[7] Centers for Disease Control and Prevention. Addressing chronic disease through community health workers: a policy and systems level approach. http://www.cdc.gov/dhdsp/docs/chw_brief.pdf. Accessed April 2, 2015.

[8] Patient Protection and Affordable Care Act, 42 USC §18001 (2010).

[9] Volkmann K , Castañares T . Clinical community health workers: linchpin of the medical home. J Ambul Care Manage 2011;34(3):221–33. 10.1097/JAC.0b013e31821cb559 21673521

[10] Ferguson WJ , Lemay CA , Hargraves JL , Gorodetsky T , Calista J . Developing community health worker diabetes training. Health Educ Res 2012;27(4):755–65. 10.1093/her/cyr080 21926065

[11] IBM SPSS Statistics. Armonk (NY): IBM Software; 2013. http://www-01.ibm.com/software/analytics/spss/products/statistics/. Accessed May 20, 2015.

[12] MAXQDA software for qualitative data analysis. Berlin (DE): VERBI Software; 2015. http://www.maxqda.com/. Accessed May 20, 2015.

[13] Guest G , Namey E , Mitchel M . Collecting qualitative data: a field manual for applied research. Washington (DC): Sage Publications; 2013.

[14] Farmer T , Robinson K , Elliott SJ , Eyles J . Developing and implementing a triangulation protocol for qualitative health research. Qual Health Res 2006;16(3):377–94. 10.1177/1049732305285708 16449687

[15] Damschroder LJ , Aron DC , Keith RE , Kirsh SR , Alexander JA , Lowery JC . Fostering implementation of health services research findings into practice: a consolidated framework for advancing implementation science. Implement Sci 2009;4(1):50. http://www.ncbi.nlm.nih.gov/entrez/query.fcgi?cmd=Retrieve&db=PubMed&list_uids=19123945&dopt=Abstract 10.1186/1748-5908-4-50 19664226 PMC2736161

[16] Wennerstrom A , Bui T , Harden-Barrios J , Price-Haywood EG . Integrating community health workers into a patient-centered medical home to support disease self-management among Vietnamese Americans: lessons learned. Health Promot Pract 2015;16(1):72–83. 10.1177/1524839914547760 25139872

[17] The Institute for Clinical and Economic Review. Community health workers: a review of program evolution, evidence effectiveness and value, and status of workforce development in New England [draft report May 24, 2013]. http://cepac.icer-review.org/wp-content/uploads/2011/04/CHW-Draft-Report-05-24-13-MASTER1.pdf. Accessed July 30, 2015.

[18] Matos S , Findley S , Hicks A , Legendre Y , Do Canto L . Paving a path to advance the community health workforce in New York State: a new summary report and recommendations. New York (NY): the New York State Community Health Worker Initiative; 2011.

[19] Crummer MB , Carter V . Critical pathways — the pivotal tool. J Cardiovasc Nurs 1993;7(4):30–7. 10.1097/00005082-199307000-00004 8326360

[20] Kapheim MG , Campbell J . Best practice guidelines for implementing and evaluating community health worker programs in health care settings. Chicago (IL): Sinai Urban Health Institute; 2014.

[21] Lemay CA , Ferguson WJ , Hargraves JL . Community health worker encounter forms: a tool to guide and document patient visits and worker performance. Am J Public Health 2012;102(7):e70–5. 10.2105/AJPH.2011.300416 22594753 PMC3477991

[22] Pittman M , Broderick A , Barnett K , Sutherland A . Bringing community health workers into mainstream of U.S. health care. Washington (DC): Institute of Medicine of the National Academies; 2015.

[23] Krieger J , Collier C , Song L , Martin D . Linking community-based blood pressure measurement to clinical care: a randomized controlled trial of outreach and tracking by community health workers. Am J Public Health 1999;89(6):856–61. 10.2105/AJPH.89.6.856 10358675 PMC1508657

[24] Centers for Disease Control and Prevention. A handbook for enhancing community health worker programs: guidance from the national breast and cervical cancer early detection program. Rockville (MD): National Training Center for the Prevention and Early Detection of Cancer; 1998.

[25] Mobula LM , Okoye MT , Boulware LE , Carson KA , Marsteller JA , Cooper LA . Cultural competence and perceptions of community health workers’ effectiveness for reducing health care disparities. J Prim Care Community Health 2015;6(1):10–5. 10.1177/2150131914540917 24986493 PMC7086339

[26] May ML , Contreras RB . Promotor(a)s, the organizations in which they work, and an emerging paradox: how organizational structure and scope impact promotor(a)s’ work. Health Policy 2007;82(2):153–66. 10.1016/j.healthpol.2006.09.002 17049668

[27] Balcázar H , Rosenthal EL , Brownstein JN , Rush CH , Matos S , Hernandez L . Community health workers can be a public health force for change in the United States: three actions for a new paradigm. Am J Public Health 2011;101(12):2199–203. 10.2105/AJPH.2011.300386 22021280 PMC3222447

[28] Rosenthal EL , Brownstein JN , Rush CH , Hirsch GR , Willaert AM , Scott JR , Community health workers: part of the solution. Health Aff (Millwood) 2010;29(7):1338–42. 10.1377/hlthaff.2010.0081 20606185

[29] Mayer M. How one medical practice integrated community health workers into the health care team. Peers for Progress [blog]; 2014. http://peersforprogress.org/pfp_blog/how-one-medical-practice-integrated-community-health-workers-into-the-health-care-team/.

[30] Brownstein JN , Hirsch GR , Rosenthal EL , Rush CH . Community health workers “101” for primary care providers and other stakeholders in health care systems. J Ambul Care Manage 2011;34(3):210–20. 10.1097/JAC.0b013e31821c645d 21673520

